# A cross-lagged panel analysis of second language achievement and enjoyment

**DOI:** 10.3389/fpsyg.2023.1046909

**Published:** 2023-02-01

**Authors:** Minjuan Gao

**Affiliations:** School of Foreign Languages, Xi’an Aeronautical Institute, Xi’an, Shaanxi, China

**Keywords:** foreign language enjoyment (FLE), actual second language (L2) achievement, English as a foreign language (EFL), new dynamic perspective, cross-lagged panel (CLP) analysis

## Abstract

Although self-perceived language proficiency has recently been found to influence foreign language enjoyment (FLE), rigorous assessment of the causal relationship between actual second language (L2) achievement and FLE has received relatively little attention. Based on control-value theory, this longitudinal study examined the causal antecedents of the relationship between the L2 achievement of 206 FL learners and their FLE from the perspective of dynamic systems theory and conducted a cross-lagged panel (CLP) analysis using Mplus 8.3 software. Both variables were measured two times over one academic year (10 months) in an English as a foreign language (EFL) course. The Wilcoxon signed-rank test showed significant changes in both variables over time. According to the CLP path model, L2 achievement at Time 1 (T1) appeared to affect subsequent FLE, while FLE at T1 failed to predict L2 achievement at Time 2 (T2). This study provides empirical evidence of the directional effect of L2 achievement on FLE regarding the hypothesized reciprocal effect of the two. Implications for stakeholders in the field of education are discussed.

## 1. Introduction

Since the integration of positive psychology into research on second language (L2) acquisition in 2012 (MacIntyre and Gregersen, 2012; Lake, 2013), there has been a greater focus on the role of positive emotions in the academic domain (Oxford, 2015). Dewaele and MacIntyre (2014) based their pioneering research on the broaden-and-build theory (Fredrickson, 2001, 2004); they examined the positive emotion of foreign language enjoyment (FLE) and negative emotion of foreign language classroom anxiety (FLCA) in L2 learners, marking an “affective turn” in applied linguistics (Lantolf and Swain, 2019). Subsequent studies advanced their research by investigating the factors underlying enjoyment (Dewaele and MacIntyre, 2016; Li et al., 2018) and dynamic fluctuations therein over time (Boudreau et al., 2018; Elahi Shirvan and Talebzadeh, 2018; Elahi Shirvan and Taherian, 2021; Elahi Shirvan et al., 2021). Predictors of enjoyment (Dewaele et al., 2016, 2018) and their effects on self-perceived foreign language performance (Dewaele and Alfawzan, 2018) have also been studied.

As a positive activity- and outcome-related achievement emotion (Pekrun, 2006; Pekrun et al., 2014), FLE has a positive relationship with L2 achievement and also mediates other desirable academic outcomes and personality traits such as achievement expectancies and trait emotional intelligence. Previous literature has revealed a significant positive relationship between FLE and students’ self-perceived language proficiency (Liu, 2013; Dewaele and Alfawzan, 2018; Botes et al., 2020; Li, 2020). Furthermore, actual academic achievement is positively related to FLE (Guo, 2021; Jin and Zhang, 2021). However, most of these studies focused on “bivariate relations” (Teimouri et al., 2019) between FLE and language achievement and did not aim to establish causality. Therefore, based on the control-value theory (CVT) (Pekrun, 2006), this longitudinal study aimed to determine whether these two variables are mutually influential (Pekrun, 2006). The study performed a cross-lagged panel (CLP) analysis, with two waves of longitudinal data collection, on students who speak English as an L2 at a university in a western province of China.

## 2. Literature review

### 2.1. Relation between enjoyment and L2 achievement

Enjoyment is characterized by a sense of novelty or accomplishment and includes eight components (Csikszentmihalyi, 1990; Seligman and Csikszentmihalyi, 2014). Research on FLE and L2 acquisition is grounded in the broaden-and-build theory of positive emotions (Fredrickson, 2001) and CVT of achievement emotions (Pekrun, 2006). The broaden-and-build theory posits that experiencing positive emotions can help people accumulate enduring personal resources, including intellectual ones. CVT argues that the interplay among various mechanisms regulates the effects of achievement emotions on academic performance (Pekrun, 2006; Shao et al., 2019). In addition, a corollary of the theory is that achievement emotions and academic achievement are mutually influential (Pekrun, 2006).

Although the relationship between FL Italian and FLE was not significant in the study by Dewaele and Proietti Ergün (2020) (*r* = 0.173, *p* < 0.073), the Pearson correlation result did show that FL English was significantly associated with FLE (*r* = 0.307, *p* < 0.001). Moreover, L2 achievement was significantly associated with FLE in three studies (Dewaele et al., 2019; Li et al., 2020; Jin and Zhang, 2021). More recently, Botes et al. (2022) conducted a meta-analysis that included 28,166 participants and reported a moderate positive correlation between FLE and L2 academic achievement (total effect size = 0.96). Li et al. (2020) found a moderate correlation between FLE and actual English achievement, and the Pearson correlation coefficient was bigger in high-achieving groups (*r* = 0.271, *p* < 0.001) compared with the entire sample of 1,307 Chinese senior high school students (*r* = 0.2, *p* < 0.001). According to the one-way analysis of variance (ANOVA) conducted in that study, learners with high English proficiency had significantly higher FLE than those with moderate and low English proficiency (both *p* < 0.001). The regression model showed that FLE for all students explained 6.9% of the variance in actual English achievement. FLE explained 18.6% of the variance in actual English achievement in the high-achieving group, and the effect size of FLE for actual English achievement was not significant in the moderate- and low-achievement groups. Moreover, FLE mediated the relationship between students’ trait emotional intelligence and English learning achievement (effect size = 0.47). Similarly, the Pearson correlation result in Guo’s (2021) study also showed that FLE was moderately correlated with academic achievement among 707 Chinese university students (*r* = 0.220, *p* < 0.0001).

### 2.2. Effect of FLE on L2 achievement

Despite efforts to explore the relationship between FLE and L2 achievement, the possible causal relationship has received little attention. However, in their cross-sectional study, Jin and Zhang (2021) performed a path analysis revealing that the “private dimension” of FLE played a more significant role in promoting L2 achievement than the “teacher dimension” and “student support dimension.” Other cross-sectional studies (Wei et al., 2019; Liu and Wang, 2021) found a significant mediating effect of FLE on the relationship between grit and L2 performance; a relationship between trait emotional intelligence and English learning achievement has also been reported (Li, 2020). In another experimental study (Kralova et al., 2022), declarative vocabulary and FLE showed a significant and concurrent post-test increase.

Relationships between learning enjoyment and loving pedagogy (Pavelescu and Petrić, 2018), teachers and teaching practices (Dewaele et al., 2018, 2022b), and wellbeing and resilience (Proietti Ergün and Dewaele, 2021), emotion regulation (Dewaele and Dewaele, 2020), academic engagement (Dewaele and Li, 2021), grit (Elahi Shirvan et al., 2021; Teimouri et al., 2022), and motivation (Dewaele et al., 2022a) have been reported.

### 2.3. Effect of L2 achievement on FLE

As causal pathways can be multi-directional (Duarte and Gogolin, 2013), possible reciprocal effects between L2 achievement and FLE have been explored. According to Dewaele and MacIntyre (2014), intermediate and advanced language learners had significantly higher FLE than low and intermediate ones, and they argued that a better command of L2 could increase global FLE. Piechurska-Kuciel (2017) reported that enjoyment of L2 was significantly stronger than that of L3 and posited that mastery of language might enhance enjoyment. Furthermore, the linear trend analysis of Botes et al. (2020) indicated that increased self-perceived FL proficiency resulted in significantly increased FLE [*F*_(1, 1,617)_ = 58.867; *p* < 0.001]. However, the statistical methods adopted in most previous studies, such as correlation and regression analyses, *t*-tests, and ANOVA, cannot establish causality (Shao et al., 2022). Similarly, the cross-sectional design of studies conducting path analysis made it difficult to determine causality (Collier, 2020), as did the non-experimental design of Dewaele et al. (2018). As such, the latter study made no claims about the extent to which FLE can predict L2 achievement. Therefore, in-depth investigations using advanced statistical procedures, other than correlation analyses, are needed to clarify causal relations (Teimouri et al., 2019; Botes et al., 2020). This longitudinal study aimed to determine whether L2 achievement affects FLE over time, whether the latter variable predicts the former one, or whether they are mutually influential.

### 2.4. Dynamic systems theory (DST): Application to second language acquisition (SLA)

As well as the integration of positive psychology into SLA, the application of DST to SLA (Larsen-Freeman and Cameron, 2008) has also increased as research on emotions has shifted toward a dynamic approach (Dewaele, 2012; Mercer and MacIntyre, 2014; Piniel and Csizér, 2015). Longitudinal designs are essential for tracking changes over time (Ortega and Iberri-Shea, 2005; Pawlak, 2012). Dewaele and Dewaele (2017) performed a pioneering pseudo-longitudinal survey to assess the dynamic interaction of FLE and anxiety in 189 foreign language students in two schools in London (UK). Longitudinal studies using more sophisticated statistical methods, such as structural equation modeling (SEM), have been applied in FLE research. Elahi Shirvan and Taherian (2021) used a latent growth curve model to longitudinally examine FLE and FLCA among university students. Elahi Shirvan et al. (2021) tracked FLE and L2 grit over 8 weeks, again using a latent growth curve model. Khajavy (2021) examined longitudinal relationships among emotions, motivation, grit, engagement, and L2 achievement through SEM. Other researchers adopted more dynamic approaches to track moment-to-moment changes in such variables (Elahi Shirvan and Talebzadeh, 2018; Elahi Shirvan et al., 2020).

According to Larsen-Freeman and Cameron (2008), two or more complex processes can change in response to each other during coadaptation; this potential reciprocal causality differs from “single causality.” Through a panel analysis of three-wave longitudinal data, Alamer and Lee (2021) examined whether L2 achievement and anxiety were mutually predictive under the moderating effect of motivation. Additionally, Alamer and Alrabai (2022) investigated the causal relationship between motivation and L2 achievement.

To test the hypothesis of a reciprocal causal relationship between L2 achievement and FLE (Botes et al., 2022), this longitudinal, empirical study performed a CLP analysis to assess the dynamic interaction between the two variables.

### 2.5. Research questions

To test the hypothesis that L2 performance and FLE interact with each other (Pekrun, 2006), this study included a CLP analysis, which is a relatively sophisticated analytical approach (Kline, 2016). In particular, this structural modeling method is better suited for analyzing dynamic interactions between two or more variables over time, and for establishing directionality in causality, than correlation and regression analyses, ANOVA, and *t*-tests, as used in previous studies.

Question 1: Do mean scores for L2 achievement and FLE change over time, and are the changes statistically significant?
Question 2: Do achievement and FLE affect each other over time?

## 3. Methodology

### 3.1. Participants

Using a convenience sampling method, 206 Chinese EFL university students (129 male students and 77 female students; 62.6 and 37.4%, respectively) aged 17–22 years (mean age = 19.54 ± 0.789 years) were recruited to this study. The participants were selected from 15 provinces and one municipality of China, including Western China (85.2%; Shaanxi, Xinjiang, Chongqing Municipality, Qinghai, Guizhou, and Gansu), Central China (9.4%; Anhui, Guangxi, Henan, Hunan, Jiangxi, and Shanxi), and Eastern China (5.4%; Guangdong, Hebei, and Liaoning). The largest proportion of students was from Shaanxi.

### 3.2. Instruments

The Chinese Version of the Foreign Language Enjoyment scale (CFLES) contains 11 positively worded items and three factors: FLE-Private, FLE-Teacher, and FLE-Atmosphere. The CFLES has been validated for use with Chinese EFL learners (Li et al., 2018). FLE-Private pertains to the FLE of Chinese EFL learners (e.g., fun, interest, and self-perceived accomplishments). FLE-Teacher relates to encouraging and positive attitudes of English teachers toward their students. FLE-Atmosphere pertains to a desirable language learning atmosphere characterized by peer support (Li et al., 2020). The CFLES uses a 5-point Likert scale ranging from “Strongly disagree” (1) to “Strongly agree” (5). Total scores range from 11 to 55 and are obtained by summing the scores for the 11 items. Previous studies (Li et al., 2018, 2021; Li, 2020) demonstrated the reliability and construct validity of the CFLES. In this study, the internal consistency was good; the composite reliability values were 0.840 and 0.895 for the first and second measurement occasions, respectively.

### 3.3. English achievement test

For Time 1 (T1; the beginning of the first semester), the English score for the National College Entrance Examination (NCEE) of 2021 was considered to reflect L2 achievement. The NCEE is an annual, nationwide standardized English test used to assess the eligibility of candidates for enrolment in Chinese universities. The maximum NCEE English test score is 150 points, and the test has shown good validity and reliability (Hu et al., 2014). The test takes 2 h and assesses the listening, reading, and writing skills of senior high school students.

For Time 2 (T2; end of the second semester of year 1), the average of the mock College English Test Band 4 and final English exam scores was used as a measure of L2 achievement. The College English Test Band 4 (total score of 750) is an annual, nationwide standardized English test for non-English majors in China that assesses students’ listening (35%), reading (35%), translation (15%), and writing (15%) abilities within 125 min. The validity and reliability of this test have also been demonstrated (Zheng and Cheng, 2008).

The final English exam is a standardized test of students’ English proficiency that takes 2 h to complete. This test assesses listening (25%), reading (40%), translation (15%), and writing (20%) abilities; it was developed by an anonymous fellow English teacher, reviewed by the Dean of the Department of English, and tested by the Office of Teaching Affairs in terms of reliability and validity.

### 3.4. Procedures

Before administering the questionnaire, the teacher/researcher obtained consent from university presidents, administrative departments, another English teacher who taught half of the participants, and all students. Data at T1 were collected in the period 7–9 September 2021. The questionnaire was administered to 206 first-year college students at the beginning of their first English class and provided information regarding their enjoyment of previous English learning activities and demographics (age, gender, class, and NCEE score). The teacher/researcher informed the participants that the demographic data would remain confidential, and also the rest of the data would only be used for research purposes. The researcher also encouraged participants to provide honest feedback on the questionnaire.

Data collection at T2 was conducted in June 2022. A College English Test-Band Four (CET-4) paper from June 2018 (the second of three), which had not been previously accessed, was randomly selected by the teaching fellow as the mock test paper. Because of the time limit of the class (100 min), writing was omitted from the final test paper (where 30 min is allocated for writing in the syllabus for CET-4).^1^ The maximum score for the mock CET-4 is 100 points; it comprises listening (35%), reading (40%), and translation (25%) components.

From 6 to 9 June 2022, the mock CET-4 was conducted during the final first-year class. The teachers did not inform the students about the content of the test beforehand so that they could objectively examine students’ overall English language proficiency and preclude the possibility of cheating. After supervising the students while setting up the mock exam, the teacher/researcher and another fellow English teacher collected the papers of 206 participants. The two teachers masked the participants’ names to avoid bias. They then calculated the students’ grades, which was a time-consuming process. The final English exam was carried out at the campus level 2 weeks later. The participants’ final exam scores were reviewed and calculated in the same way. At the end of the academic year (in July), the same questionnaire was administered online to the same group of students^2^.

### 3.5. Data analyses

Regarding missing data, the non-response rates for variables “FLE” and “L2 achievement” were 10.2% and 7.3% for the first wave of the survey, respectively; for the second wave, the respective rates were 1% and 0%. Multiple imputations were used to handle missing values; this is superior to the conventional approach because it accounts for non-normal data and data missing at random (Enders, 2010; Little and Rubin, 2014; Shi et al., 2020). The same teachers were involved in both waves of the survey and there were no outliers in the dataset. The normality of the data distribution was determined based on skewness and kurtosis cut-off values suggested by Collier (2020) and the results of the Shapiro–Wilk test.

The mean data for the first research question were analyzed using the Wilcoxon signed-rank test, performed using SPSS software (ver. 23.0; IBM Corp., Armonk, NY, USA). This test was chosen in preference to the paired sample *t*-test because the data did not show a normal distribution (Table 1). For the second research question, Spearman correlation analysis was used to analyze relationships among variables; this was also performed using SPSS (ver. 23.0). Then, confirmatory factor analysis (CFA) was conducted using Mplus 8.3 software (Muthén and Muthen, 2017) to determine if the measurement model fit the CFLES data well. Several model fit indices were used, including the Chi-square statistic (χ^2^) comparative fit index (CFI), Tucker–Lewis index (TLI), root mean square error of approximation (RMSEA) and its 90% confidence interval, and standardized root mean residual (SRMR). Finally, CLP analysis was performed to ascertain the directionality in the relationship between the two variables over time (Kline, 2016). Similar to the CFA, model fit indices were calculated. Bias-corrected bootstrap confidence intervals (5,000 bootstraps) were calculated because of the non-normal distribution of some of the data (Kline, 2016; Muthén and Muthen, 2017). For both the measurement model and CLP model, maximum likelihood estimation was performed (Alamer and Alrabai, 2022).

**TABLE 1:** Spearman correlations of the study variables.

	1	2	3	4
1. L2 achievement at T1	1			
2. FLE at T1	0.289**	1		
3. L2 achievement at T2	0.454**	0.242**	1	
4. FLE at T2	0.229**	0.329**	0.356**	1
Skewness	-0.172	-0.03	-0.270	0.830
Kurtosis	-0.420	2.921	0.406	3.169
Mean	62.090	40.425	70.568	41.738
Standard deviation	13.31907	6.82	9.91	6.786

**p < 0.01.

## 4. Results

### 4.1. Descriptive statistics

The variables “FLE at T1” and “FLE at T2” exceeded the cut-off values of +2 and −2, respectively (Collier, 2020). The Shapiro–Wilk test showed that none of the variables conformed to a normal distribution (L2 achievement at T1, *W* = 0.991, *p* < 0.05; FLE at T1, *W* = 0.975, *p* < 0.05; L2 achievement at T2, *W* = 0.993, *p* < 0.05; and FLE at T2, *W* = 0.983, *p* < 0.05).

According to Spearman correlation analysis, L2 achievement at T1 was moderately correlated with FLE at T1 (*r* = 0.289, *p* < 0.01). Similarly, L2 achievement at T2 was moderately correlated with FLE at T2 (*r* = 0.356, *p* < 0.01). These results indicate that the correlation strength between the two variables was stable over 10 months. The association between L2 achievement at T1 and T2 was also moderate (*r* = 0.454, *p* < 0.01), similar to FLE at T1 and FLE at T2 (*r* = 0.329, *p* < 0.01) (Table 1).

The score for mean L2 achievement at T2 (70.568 ± 9.91) was 8.478 points higher than that for L2 achievement at T1 (62.090 ± 13.319). The mean score for global FLE at T2 (41.738 ± 6.786) was higher than that for FLE at T1 (40.425 ± 6.82). According to the Wilcoxon signed-rank test, the median score for L2 achievement at T2 (71) was significantly higher than that for L2 achievement at T1 (61.3; *Z* = −21.055, *p* < 0.001). Furthermore, the median score for FLE at T2 (41) was significantly higher than that for FLE at T1 (40; *Z* = −4.558, *p* < 0.001).

The mean score for L2 achievement at T2 (70.568 ± 9.91) for the entire sample was significantly higher than that for L2 achievement at T1 (62.090 ± 13.319). Similarly, the mean score for FLE at T2 (41.738 ± 6.786) was significantly higher than that for FLE at T1 (40.425 ± 6.82).

### 4.2. Structural model

The goodness-of-fit indices of the measurement model (CFLES) indicated a good fit to the data for both the first measurement occasion [χ^2^ = 390.069, degrees of freedom *df* = 41, *p* = 0, CFI = 0.950, TLI = 0.933, RMSEA = 0.084 (90% CI: 0.076, 0.091), SRMR = 0.048] and second measurement occasion [χ^2^ = 585.349, *df* = 41, *p* = 0, CFI = 0.947, TLI = 0.929, RMSEA = 0.104 (90% CI: 0.096, 0.111), SRMR = 0.050].

The CLP model was shown to be a saturated model by the goodness-of-fit index (χ^2^ = 0, *df* = 0) and explained 19.7% of the variance in L2 achievement (*R*^2^ = 0.197) and 15.8% of that in FLE (R^2^ = 0.158) at T2.

**FIGURE 1:**
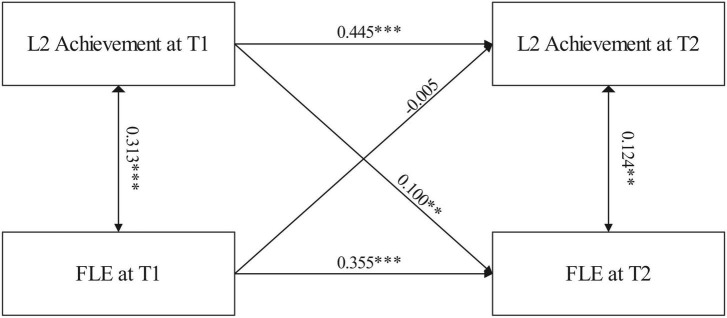
Results of the cross-lagged panel analysis. ^**^p < 0.01, ^***^p < 0.001.

For the CLP model, the correlation between L2 achievement and FLE for both measurement occasions was moderate but significant at T1 (*r* = 0.313, *p* < 0.001) and weak but significant at T2 (*r* = 0.124, *p* < 0.01). The effect of students’ L2 achievement at T1 on T2 was moderate (β = 0.445, *p* < 0.001), as was that of the students’ FLE (β = 0.355, *p* < 0.001) (Figure 1). The strength of the path coefficients accorded with the most recent L2 guidelines (Hair and Alamer, 2022).

The results for the time-lagged paths between L2 achievement T3 and FLE revealed that the initial L2 achievement significantly affected FLE at T2, although the path coefficient was small (β = 0.1, *p* < 0.01). No reciprocal effect was observed, however; FLE in the initial phase did not influence L2 at T2 (β = −0.005, *p* = 0.898).

## 5. Discussion

Theoretical works on the reciprocal relationships of academic achievement with various environmental, cognitive, and behavioral factors (Calsyn and Kenny, 1977; Bandura, 1978; Bandura et al., 1999) paved the way for empirical studies seeking to establish causality between self-efficacy and achievement (Williams and Williams, 2010; Hwang et al., 2016; Schöber et al., 2018) and self-concept and achievement (Huang, 2011; Retelsdorf et al., 2014; Schöber et al., 2015), for example. In contrast, the reciprocal relationship between L2 achievement and FLE is still an emerging field of research (Shao et al., 2019). Therefore, this study sought to establish the direction of the relationship between these two variables, where Pekrun et al. (2014) postulated that outcome-related achievement emotions are dependent on academic achievement and numerous other factors.

This study extended the extant literature by rigorously examining the reciprocal relationship between L2 achievement and FLE. Regarding the first research question, the Wilcoxon signed-rank test revealed that the mean score for L2 achievement at T2 was significantly higher than that of L2 achievement at T1. Similarly, the mean score for FLE at T2 was significantly higher than that of FLE at T1. Therefore, both variables changed significantly over time. The second question explored whether changes in these two complex systems are interrelated. The moderate correlations between L2 achievement and FLE at T1 and T2 accord with previous studies (Dewaele et al., 2019; Dewaele and Proietti Ergün, 2020; Li, 2020; Li et al., 2020; Guo, 2021; Jin and Zhang, 2021). Although a direct correlation was found between L2 achievement and FLE, the CLP analysis in this study suggested that the relationship between these variables changed over time. Earlier L2 achievement had a weak effect on FLE at T2 and the evidence for a reciprocal effect was inconclusive. This finding echoes that of Alamer and Alrabai (2022), who found that L2 achievement at T2 affected autonomous motivation at Time 3 (T3), while motivation at T2 failed to predict achievement at Time 3. These results imply that learners’ achievements before entering college contribute to FLE at the end of the freshman semester, whereas the opposite does not appear to be the case. This finding was not in line with our expectations but does suggest that L2 achievement is an antecedent of FLE. This result may be explained by contingent path theory (Raynor and Roeder, 1987) and the theory of psychological momentum (Guenther and Kokotajlo, 2017; Hubbard, 2017), which posits that desirable learning task outcomes enhance motivation to pursue learning and undertake further tasks. This is supported by the self-determination theory, where one study found that self-perceived competence enhances students’ motivation during subsequent L2 learning (Alamer, 2022). Similarly, academic achievement enhances students’ enjoyment of L2 learning and enables them to overcome future obstacles. As proficiency and mastery increase, the enjoyment of students also increases.

Although the effect of earlier L2 achievement on subsequent FLE in this study was not strong, it was nevertheless statistically significant and highlighted the importance of L2 mastery to FLE as students mature, as well as to the accrual of resources and ability to further advance. The lack of a strong effect also points to the necessity to integrate emotion, motivation, cognition, and L2 achievement into models to uncover the mechanism through which positive emotions and achievement mutually reinforce each other through a virtuous cycle.

In this study, the retest reliability of L2 achievement (*r* = 0.454, *p* < 0.001) and FLE (*r* = 0.329, *p* < 0.001) were moderate rather than high, which could be explained by the long interval (10 months) between the two measurement occasions compared with previous studies, namely, 6 weeks (Alamer and Lee, 2021; Alamer and Alrabai, 2022) and 2 weeks (Elahi Shirvan et al., 2021). Moreover, the three standardized tests used in this study as indicators of L2 achievement (the NCEE, mock CET-4, and final English exam) differ in difficulty; the mock CET-4 is more difficult than the other two. Therefore, the direction of the relationship between FLE and L2 achievement should be inferred with caution; additional longitudinal studies and cross-legged panel analyses are required.

## 6. Limitations and implications

Although the participants in this study did not exhibit homogeneity in terms of demographic characteristics, unlike previous studies, they were nonetheless recruited from the same provincial-level university and were of similar age. Notably, the majority of the participants were from Shaanxi and thus are not representative of all EFL learners at Chinese universities. Furthermore, the modest sample size and non-normal data distribution may make it more difficult to obtain significant results. Although bias-corrected bootstrap confidence intervals were calculated to avoid bias, the results of the CLP path model should be interpreted with caution. In addition, as the study period was only 10 months, data for the sophomore year (which is 10 months after the second measurement occasion), junior year, and senior year were not available for analysis. Fluctuations in the mutual relationship between FLE and achievement remain to be investigated at both short- (weekly) and long-term (up to the point of degree completion) timeframes. Finally, as this quantitative study used a self-report questionnaire, the data for some of the FLE dimensions, such as FLE-Teacher and FLE-Atmosphere, may have been susceptible to bias. Obtaining data for these measures from other stakeholders in the L2 classroom, including other English teachers, would be interesting.

This study has three main implications. First, future research on the causal relationship between L2 achievement and FLE should use a mixed-methods approach to validate the effect of previous learning achievement on future FLE (Alamer and Alrabai, 2022). Moreover, additional research on the mechanism through which L2 achievement affects FLE is necessary, including exploration of possible mediating factors; panel analyses of variables such as gender and motivation would be useful to that end. Second, L2 instructors should focus on helping learners make progress in the early stages of language learning to enhance their FLE, which may in turn help them achieve better academic outcomes. Third, L2 learners should engage in instructional activities and learning tasks both inside and outside of the L2 classroom, to accelerate their progress, enhance FLE, and motivate them to overcome difficulties during their L2 language journey.

## 7. Conclusion

This longitudinal study advances our understanding of the causal relationship between language achievement and FLE, given the relative lack of empirical evidence of any such relationship and the predominance of cross-sectional studies in the literature. The Wilcoxon signed-rank test indicated that both variables changed significantly over time, while the CLP analysis supported a causal relationship between earlier L2 achievement and subsequent FLE. Therefore, initial L2 success appears important for FLE in L2 learners. However, given the seemingly counterintuitive finding of a statistically non-significant effect of FLE on L2 achievement, further panel analyses are required.

## Data availability statement

The original contributions presented in this study are included in this article/supplementary material, further inquiries can be directed to the corresponding author.

## Author contributions

The author confirms being the sole contributor to this work and has approved it for publication.

## Footnotes

1
https://cet.neea.edu.cn/html1/folder/16113/1588-1.htm

2
https://www.wjx.cn/
